# Association of red blood cell transfusions with periventricular leukomalacia in very preterm infants

**DOI:** 10.1111/vox.70239

**Published:** 2026-03-12

**Authors:** Thomas Brune, Christian Weise, Siegfried Kropf

**Affiliations:** ^1^ Children's Hospital University Hospital Magdeburg Magdeburg Germany; ^2^ Institute for Medical Data Science University Hospital Magdeburg Magdeburg Germany; ^3^ Present address: Children's Hospital University Hospital Bonn Center of Paediatrics Bonn Germany

**Keywords:** neuropathology, preterm, red blood cell transfusion

## Abstract

**Background and Objectives:**

Red blood cell (RBC) transfusions have been associated with retinopathy of prematurity (ROP) and adverse long‐term neurodevelopmental outcomes in very preterm infants. Although lower transfusion thresholds reduce the incidence of ROP, they have not improved neurological outcome. Periventricular leukomalacia (PVL) is a major cause of long‐term neurodevelopmental impairment in very preterm infants. The objective of this study was to determine whether exposure to RBC transfusions is an independent, dose‐dependent risk factor for PVL.

**Materials and Methods:**

A retrospective cohort study was conducted. Infants born at 32 weeks of gestation or less or with a birth weight <1500 g were eligible. Infants transferred from other hospitals, managed with palliative care or who died before cranial imaging were excluded. Exposure was defined as the cumulative number of RBC transfusions during neonatal intensive care. Outcomes were PVL and severe ROP. Multivariable logistic regression analyses were performed and stratified by birth weight <750 g and ≥750 g.

**Results:**

RBC transfusions were independently associated with PVL and ROP. The adjusted odds ratio was 1.10 and 1.09, respectively, in infants weighing <750 g, and 1.26 and 1.15 in infants weighing 750 g or more, respectively, with statistically significant confidence intervals.

**Conclusion:**

RBC transfusions were independently associated with PVL in preterm infants. As lowering transfusion thresholds has not been associated with improved neurological outcomes, consideration of cord blood products may be warranted.

**Highlights**

- Cumulative exposure to adult donor red blood cell (RBC) transfusions was independently associated with periventricular leukomalacia (PVL) in very preterm infants, with a dose‐dependent relationship across birth weight strata.
- Associations between RBC transfusions and severe retinopathy of prematurity were also observed but were smaller and less consistent than those observed for PVL.
- PVL may represent a product‐sensitive neurological outcome parameter in transfusion research and may be relevant for future studies evaluating alternative RBC products.

## INTRODUCTION

Anaemia of prematurity is a frequent complication of very preterm birth and is associated with impaired oxygen delivery, increased cerebral circulatory stress and an elevated risk of hypoxic–ischaemic brain injury [1, 2, 3]. Compensatory mechanisms, including increased cerebral blood flow and oxygen extraction, may temporarily preserve cerebral oxygenation but can themselves contribute to vascular instability and brain injury when anaemia becomes severe or prolonged [4, 5, 6].

Red blood cell (RBC) transfusion represents a cornerstone of neonatal intensive care and reliably improves short‐term physiological parameters, including oxygen‐carrying capacity and cerebral oxygenation [7, 8, 9, 10]. These short‐term effects have been consistently demonstrated in physiological and near‐infrared spectroscopy studies. However, short‐term stabilization does not necessarily translate into improved long‐term neurological outcomes [11].

Despite these short‐term benefits, several studies have reported associations between RBC transfusions and adverse long‐term outcomes in preterm infants, including higher incidences of retinopathy of prematurity (ROP), necrotizing enterocolitis, bronchopulmonary dysplasia and impaired neurodevelopmental outcomes [12, 13]. In particular, for ROP, exposure to adult donor RBC transfusions has been shown to represent an independent risk factor in multivariable analyses [14, 15].

In response to these observations, transfusion guidelines have been revised in many countries, leading to the adoption of more restrictive transfusion strategies. Randomized studies have shown a trend that lower transfusion thresholds reduce the incidence of ROP, but they have not demonstrated consistent improvements in intraventricular haemorrhage (IVH)/periventricular leukomalacia (PVL) and/or long‐term neurological outcome [16, 17, 18, 19, 20]. These findings suggest that threshold‐based strategies may be insufficient to modify cerebral injury.

In major randomized trials, severe IVH and cystic PVL were combined into a single neurological endpoint to increase robustness and statistical power. However, IVH and PVL differ fundamentally in their underlying pathophysiology, timing and sensitivity to disturbances in oxygen exposure and perfusion. IVH primarily reflects early mechanical and hemodynamic instability, whereas PVL represents ischaemic–inflammatory white matter injury, which is closely linked to oxygen delivery and microvascular perfusion. Historically, ROP and PVL were the two clinical entities that triggered the introduction of oxygen‐targeting strategies in preterm infants, reflecting their high sensitivity to disturbances in oxygen exposure and distribution. PVL, a hallmark of cerebral white matter injury in preterm infants, has shown inconsistent associations with transfusion thresholds across randomized trials and has generally been analysed as a secondary outcome without consideration of cumulative transfusion exposure [16, 17, 18, 19, 21]. As a result, it remains unclear whether PVL represents a threshold‐dependent phenomenon or a product‐related, dose‐dependent adverse effect of adult RBC transfusion.

PVL is routinely assessed during neonatal care using standardized cranial ultrasound and represents an early, organ‐specific marker of brain injury that is not subject to follow‐up bias [17, 18]. Evaluating PVL in relation to cumulative transfusion exposure may therefore provide insights into transfusion‐associated neurological risk that are not captured by transfusion threshold comparisons alone. In the present study, we investigate whether exposure to adult RBC transfusions is independently associated with PVL and compare this relationship with the effect on ROP.

## MATERIALS AND METHODS

### Study design and data source

We performed a retrospective cohort study using prospectively collected data from the German Neonatal Network (GNN), a nationwide observational registry of very preterm infants. GNN records perinatal and neonatal risk factors, clinical outcomes and selected genetic data from Level 1 perinatal centres across Germany. For the present analysis, we used the dataset from 2009 to 2015, which included 12,250 infants. The study period (2009–2015) was chosen to ensure a homogeneous cohort with stable transfusion practices and complete standardized cranial ultrasound follow‐up across participating GNN centres.

### Participants

Eligible were infants born ≤32 weeks' gestation or with a birth weight <1500 g whose parents provided informed consent. For the present analysis, we included only inborn infants not managed with primary or secondary palliative care. Infants who died before cranial imaging could be performed were excluded. Implausible or inconsistent entries (e.g., implausible oxygen therapy durations, missing values for end of respiratory support) were corrected according to predefined rules. Because the impact of prematurity on vascular and neuronal development is particularly pronounced at a birth weight <750 g or <26 weeks' gestation, infants were stratified into two subgroups: <750 g versus ≥750 g.

### Exposure

The primary exposure was the cumulative number of RBC transfusion units administered during the initial hospitalization. Other blood products (fresh frozen plasma, platelets) were not included.

### Outcomes

The primary outcome was PVL, which was diagnosed by serial cranial ultrasound according to standardized definitions used within the GNN, consistent with previously published GNN methodology [22].

PVL reflects clinically relevant periventricular white matter injury diagnosed by experienced neonatologists and paediatric radiologists in participating Level 1 centres. Early transient periventricular echogenicity alone is not considered diagnostic unless followed by persistent or cystic white matter changes on serial imaging. Differentiation from physiological immaturity and PVH is part of routine clinical practice. Isolated transient periventricular echogenicity without subsequent cystic or persistent changes was not classified as PVL.

Magnetic resonance imaging (MRI) was not routinely required for registry documentation; therefore, PVL reflects clinically relevant, sonographically diagnosed white matter injury. This approach is consistent with established descriptions of cystic PVL in preterm infants [17].

The secondary outcome was severe ROP, defined as stage ≥3 according to the International Classification of ROP. For comparability with previous studies, stages 3–5 were grouped as ‘severe ROP’ (sROP).

### Covariates

Prespecified covariates included gestational age, birth weight, sex, mode of delivery, multiple birth, 10‐min Apgar score, oxygen exposure (days of supplemental O_2_, maximum FiO_2_ in first 12 h), end of respiratory support, culture‐proven sepsis and erythropoietin treatment.

### Statistical analysis

Candidate risk factors were first evaluated in bivariate analyses (*χ* ^2^ test for categorical variables; Mann–Whitney *U* test for continuous variables). All variables with *p* < 0.05 in univariate testing were considered candidates.

For the multivariate models, only complete datasets were analysed, which led to different sample sizes depending on the number of covariates included. A stepwise forward logistic regression was then performed separately for both weight strata (<750 g and ≥750 g). Initially, all prespecified covariates were entered as a full model to assess their joint contribution and identify potential predictors. Stepwise forward selection was performed to identify the subset of predictors that remained independently associated with the outcomes, whereby variables that did not significantly improve model fit were excluded. This procedure reduced the number of predictors and simultaneously increased the number of infants available for analysis. The sequence of variable inclusion and exclusion is documented in Supporting Information.

Three models were fitted:

1. Full model including all prespecified covariates.
2. Stepwise forward selection model, retaining only independently significant predictors.
3. Reduced 5‐variables model (RBC transfusions, gestational age, birth weight, oxygen exposure, sepsis) to maximize sample size in the presence of missing data.

All analyses were stratified by birth weight (<750 g vs. ≥750 g). Odds ratios (ORs) with 95% confidence intervals (CIs) were reported. Statistical significance was defined as *p* < 0.05 (two‐sided). Analyses were conducted using SPSS Statistics (IBM, version 24).

### Ethics statement

The GNN study was approved by the ethics committees of all participating centres. Written informed consent was obtained from parents or guardians.

## RESULTS

### Patient characteristics

A total of 12,250 very preterm infants were enrolled in the GNN. Of these, 2085 (17%) had a birth weight <750 g and 10,165 (83%) had a birth weight ≥750 g. Valid information on ROP stage was available for 7975 infants (65.1%), while PVL status was documented in 12,198 infants (99.6%). Severe ROP (stages 3–5) occurred in 436 infants (3.6%), and PVL was diagnosed in 225 infants (1.8%). Baseline characteristics are summarized in Table 1.

**TABLE 1:** Baseline characteristics of study cohort (German Neonatal Network, 2009–2015).

Characteristics	<750 g (*n* = 2085)	≥750 g (*n* = 10,165)	Total (*n* = 12,250)
Gestational age, mean (SD), weeks	25.6 (1.7)	29.8 (2.4)	29.0 (2.7)
Birth weight, mean (SD), g	615 (102)	1192 (219)	1095 (274)
Male sex, *n* (%)	1130 (54.2)	5040 (49.6)	6170 (50.4)
Multiple birth, *n* (%)	520 (24.9)	3540 (34.8)	4060 (33.1)
Mode of delivery: Spontaneous	480 (23.0)	2360 (23.2)	2840 (23.2)
Mode of delivery: Caesarean (elective)	1220 (58.5)	5750 (56.6)	6970 (56.9)
Mode of delivery: Caesarean (emergency)	385 (18.5)	2055 (20.2)	2440 (19.9)
10‐min Apgar score, mean (SD)	8.1 (1.2)	8.7 (1.0)	8.6 (1.1)
Days on supplemental O_2_, median (IQR)	55 (20–90)	10 (0–30)	15 (0–40)
Max. FiO_2_ in first 12 h, mean (SD), %	47 (25)	36 (20)	38 (22)
Days on respiratory support, median (IQR)	70 (40–110)	22 (10–40)	30 (15–50)
RBC transfusions, median (IQR)	4 (2–6)	1 (0–2)	2 (0–3)
Sepsis, *n* (%)	1130 (54.2)	2150 (21.2)	3280 (26.8)
EPO prophylaxis, *n* (%)	310 (14.9)	910 (9.0)	1220 (10.0)
Severe ROP (stage 3–5), *n* (%)	275 (13.2)	161 (1.6)	436 (3.6)
PVL, *n* (%)	77 (3.7)	148 (1.5)	225 (1.8)

*Note*: Values are expressed as number (percentage) unless otherwise specified.

Abbreviations: EPO, erythropoietin; IQR, interquartile range; O_2_, oxygen; PVL, periventricular leukomalacia; RBC, red blood cell; ROP, retinopathy of prematurity.

### Association of transfusions with PVL

In multivariable regression (Table 2), RBC transfusions remained independently associated with PVL in both weight strata. The odds of PVL increased by 10% per transfusion in infants <750 g (adjusted odds ratio [aOR] 1.10; 95% CI: 1.06–1.14; *p* < 0.001) and by 26% per transfusion in infants ≥750 g (aOR 1.26; 95% CI: 1.22–1.30; *p* < 0.001).

**TABLE 2:** Adjusted odds ratios for periventricular leukomalacia and retinopathy of prematurity by birth weight stratum.

Outcome	Risk factor	<750 g, aOR (95% CI); *p*	≥750 g, aOR (95% CI); *p*
PVL	RBC transfusions (per unit)	1.10 (1.06–1.14); <0.001	1.26 (1.22–1.30); <0.001
	Gestational age (per week)	NS	0.76 (0.68–0.85); <0.001
	Sepsis	1.94 (1.10–3.40); 0.020	1.68 (1.20–2.30); 0.003
	10‐min Apgar score (per point)	NS	0.77 (0.65–0.91); 0.001
	Birth weight (per 100 g)	NS	1.02 (1.01–1.04); 0.010
ROP	RBC transfusions (per unit)	1.09 (1.06–1.13); <0.001	1.15 (1.10–1.20); <0.001
	Gestational age (per week)	0.82 (0.75–0.90); <0.001	0.63 (0.55–0.70); <0.001
	O_2_ days (per day)	1.01 (1.00–1.02); 0.040	1.02 (1.01–1.03); <0.001
	Sepsis	NS	NS

*Note*: Data are aORs with 95% CIs and corresponding *p*‐values from multivariable logistic regression analyses. Models were stratified by birth weight (<750 g and ≥750 g).

Abbreviations: aOR, adjusted odds ratio; CI, confidence interval; NS, not significant; O_2_, oxygen; PVL, periventricular leukomalacia; RBC, red blood cell; ROP, retinopathy of prematurity.

In the ≥750 g stratum, additional independent predictors included sepsis (aOR 1.68; 95% CI: 1.20–2.30; *p* = 0.003) and higher 10‐min Apgar score, which was associated with lower PVL risk (aOR 0.77 per point; 95% CI: 0.65–0.91; *p* = 0.001).

### Association of transfusions with severe ROP

In bivariate analyses, RBC transfusions were associated with severe ROP in both strata. In multivariable regression (Table 2), each additional RBC transfusion increased the odds of severe ROP by 9% in infants <750 g (aOR 1.09; 95% CI: 1.06–1.13; *p* < 0.001) and by 15% in infants ≥750 g (aOR 1.15; 95% CI: 1.10–1.20; *p* < 0.001).

Gestational age was inversely associated with severe ROP in both groups (aOR 0.82 per week for <750 g and 0.63 per week for ≥750 g; both *p* < 0.001). Oxygen exposure was independently associated with increased ROP risk.

### Birth weight stratification

Across both predefined birth‐weight strata (<750 g and ≥750 g), RBC transfusions were associated with PVL and severe ROP in both bivariate and multivariable analyses.

## DISCUSSION

In this large multicentre cohort, RBC transfusions remained independently associated with PVL as well as with severe ROP in very preterm infants. Although the ORs per transfusion were modest, their cumulative impact is clinically relevant given that many very preterm infants receive multiple transfusions during neonatal care.

Anaemia of prematurity is common in very preterm infants and is associated with impaired oxygen delivery and increased cerebral circulatory stress, which may predispose to hypoxic–ischaemic brain injury [1, 2, 3, 4]. Compensatory increases in cerebral blood flow and oxygen extraction can preserve tissue oxygenation in the short term but may themselves contribute to vascular instability and brain injury when anaemia is severe or prolonged [3, 4, 5, 6]. RBC transfusion reliably improves oxygen‐carrying capacity and cerebral oxygenation, as demonstrated by physiological and near‐infrared spectroscopy studies [4, 5, 6, 7]. However, short‐term improvements in cerebral oxygenation do not necessarily translate into improved long‐term neurological outcomes.

Previous studies have primarily focused on ROP and IVH as transfusion‐associated outcomes [9, 10, 11]. Consistent with earlier reports, we observed an association between transfusion exposure and severe ROP. However, the association with PVL was more consistent and numerically stronger. PVL represents a direct marker of cerebral white matter injury and is closely linked to adverse long‐term neurodevelopmental outcomes, including cerebral palsy and cognitive impairment [17, 18]. By including PVL as a predefined endpoint, our study expands the assessment of transfusion‐related neurological risk beyond retinal outcomes.

Randomized controlled trials comparing liberal and restrictive transfusion thresholds, including PINT, TOP and ETTNO, have demonstrated a trend towards reduction in ROP with lower thresholds but have not shown consistent differences in survival or major neurodevelopmental outcomes between strategies [12, 13, 14, 15]. These findings suggest that threshold‐based approaches alone may be insufficient to modify cerebral injury.

Two non‐exclusive mechanisms may contribute to transfusion‐associated long‐term damages. First, adult donor erythrocytes differ substantially from neonatal RBCs with respect to deformability, size and oxygen‐binding characteristics [19, 20]. These differences may impair microvascular perfusion in vulnerable capillary beds, including the immature cerebral and retinal circulation. Experimental and modelling studies have shown that extremely preterm infants have particularly narrow capillary diameters, increasing susceptibility to rheological disturbances when exposed to less deformable adult erythrocytes [19, 20]. In our cohort, associations between transfusion exposure and PVL or ROP were observed across birth‐weight strata, suggesting that vulnerability to transfusion‐related microvascular effects is not confined to the most immature infants.

Second, RBC transfusions in very preterm infants are accompanied by a rapid decline in foetal haemoglobin and a corresponding increase in adult haemoglobin, resulting in altered oxygen dissociation characteristics and tissue oxygen delivery [21, 22]. Lower ratios of foetal to adult haemoglobin have been associated with an increased risk of ROP [10, 11]. Taken together, these observations support the concept that modifying transfusion thresholds alone may be insufficient and that the characteristics of the transfused product itself may be of critical importance.

The current transfusion practice exposes very preterm infants to adult donor erythrocytes that differ fundamentally from neonatal RBCs in size, deformability and haemoglobin composition, resulting in a mismatch between transfused cell properties and the physiological requirements of the immature cerebral microcirculation.

Strategies aimed at reducing exposure to adult haemoglobin A donor blood, including delayed cord clamping and improved anaemia prevention, have therefore received increasing attention. Autologous haemoglobin F cord blood transfusion has been shown to be feasible and safe in preterm infants [23, 24]. To date, the clinical use of allogeneic cord blood–derived erythrocytes has been limited, with neonatal experience largely restricted to small feasibility and safety studies from Europe and India [25, 26, 27].

The blood transfusions from umbilical cord blood to neonates study represents an important step towards haemoglobin F transfusion strategies by demonstrating that allogeneic cord blood red cell transfusion can significantly reduce the incidence of severe ROP [28, 29]. However, the study was not designed to assess early neurological effects of transfusion, as neurological outcomes were evaluated exclusively through long‐term follow‐up. As a consequence, potential transfusion‐related effects on early cerebral injury could not be addressed within that framework.

While long‐term neurodevelopmental assessment remains essential, reliance on late outcomes alone limits the feasibility and interpretability of interventional trials targeting transfusion biology. PVL is routinely assessed during neonatal care using standardized cranial ultrasound, reflects early cerebral white matter injury and is temporally aligned with transfusion exposure [17, 18]. Our findings suggest that PVL may serve as a complementary early neurological outcome parameter alongside ROP in future studies evaluating transfusion‐based strategies, including haemoglobin‐preserving approaches.

Strengths of this analysis include the large sample size, multicentre design, standardized data collection within the GNN and near‐complete ascertainment of PVL. Limitations include the retrospective design, incomplete documentation of ROP, lack of detailed information on transfusion triggers and volumes and potential residual confounding. In addition, PVL diagnosis relied primarily on cranial ultrasound, which may underestimate diffuse white matter injury compared with MRI [17].

In conclusion, in this large multicentre cohort of very low birth weight infants, exposure to adult RBC transfusions was independently associated with the occurrence of both PVL and severe ROP. Importantly, a clear dose‐dependent relationship was observed for both outcomes, indicating that cumulative transfusion exposure represents a relevant biological factor rather than a purely dichotomous treatment variable.

Although causality cannot be proven in a retrospective analysis, the consistency, strength and dose dependency of these associations support their biological plausibility and are consistent with epidemiological patterns typically observed in biologically plausible exposure–outcome relationships [30]. Transfusion‐related alterations in oxygen transport characteristics and microvascular perfusion may therefore contribute to injury of vulnerable neural and retinal tissue in very preterm infants.

These findings remain clinically relevant even in the context of increasingly restrictive transfusion strategies. Emerging approaches that aim to preserve physiological oxygen transport properties may therefore warrant further prospective evaluation. Given the potential long‐term neurodevelopmental consequences of PVL and severe ROP, a more differentiated view of transfusion exposure, including cumulative dose and qualitative aspects of transfused blood, such as foetal haemoglobin–rich cord blood–derived RBC products, appears justified.

## CONFLICT OF INTEREST STATEMENT

The authors declare no conflicts of interest.

## Supporting information


**Table S1.** Valid datasets for periventricular leukomalacia: Number of valid and missing periventricular leukomalacia datasets in the German Neonatal Network cohort.
**Table S2a**. Chi^2^ tests (<750 g): Results of *χ* ^2^ tests for categorical variables in infants <750 g BW.
**Table S2b**. Chi^2^ tests (≥750 g): Results of *χ* ^2^ tests for categorical variables in infants ≥750 g BW.
**Table S3a**. Mann–Whitney *U* tests (<750 g): Results of Mann–Whitney *U* tests for continuous variables in infants BW < 750 g BW.
**Table S3b**. Mann–Whitney *U* tests (≥750 g): Results of Mann–Whitney *U* tests for continuous variables in infants **≥**750 g BW.
**Table S4**. Distribution of variables. Number of complete datasets available for the full logistic regression model.
**Table S5**. Logistic regression (full model): Results of the full model including all prespecified covariates.
**Table S6a**. Available datasets stepwise: Number of complete datasets available for stepwise forward regression.
**Table S6b**. Results stepwise (<750 g/≥750 g): Results of the stepwise forward regression in both weight strata.
**Table S7a**. Available datasets reduced model: Results of the reduced model including five key predictors (red blood cell transfusions, gestational age, birth weight, oxygen exposure, sepsis).
**Table S7b**. Results reduced model (<750 g/≥750 g): Results of the reduced model including five key predictors (red blood cell transfusions, gestational age, birth weight, oxygen exposure, sepsis).

**Supplementary 2** German Neonatal Network (GNN) Collaborators (2009–2015).

## Data Availability

The data that support the findings of this study are available on request from the corresponding author. The data are not publicly available due to privacy or ethical restrictions.
