# Supplementary material for: Association of red blood cell transfusions with periventricular leukomalacia in very preterm infants
Source: Vox Sang. 2026 Mar 12;121(6):842–7. doi: 10.1111/vox.70239 (PMC13253060; doi:10.1111/vox.70239)
Supplement: Supplementary file 1 — Table S1. Valid datasets for periventricular leukomalacia: Number of valid and missing periventricular leukomalacia datasets in the German Neonatal Network cohort. Table S2a. Chi2 tests (<750 g): Results of χ 2 tests for categorical variables in infants <750 g BW. Table S2b. Chi2 tests (≥750 g): Results of χ 2 tests for categorical variables in infants ≥750 g BW. Table S3a. Mann–Whitney U tests (<750 g): Results of Mann–Whitney U tests for continuous variables in infants BW < 750 g BW. Table S3b. Mann–Whitney U tests (≥750 g): Results of Mann–Whitney U tests for continuous variables in infants ≥750 g BW. Table S4. Distribution of variables. Number of complete datasets available for the full logistic regression model. Table S5. Logistic regression (full model): Results of the full model including all prespecified covariates. Table S6a. Available datasets stepwise: Number of complete datasets available for stepwise forward regression. Table S6b. Results stepwise (<750 g/≥750 g): Results of the stepwise forward regression in both weight strata. Table S7a. Available datasets reduced model: Results of the reduced model including five key predictors (red blood cell transfusions, gestational age, birth weight, oxygen exposure, sepsis). Table S7b. Results reduced model (<750 g/≥750 g): Results of the reduced model including five key predictors (red blood cell transfusions, gestational age, birth weight, oxygen exposure, sepsis). [file VOX-121-842-s001.docx]

**Supplement 1. Statistics**

***Additional statistical analyses on periventricular leukomalacia (PVL) and retinopathy of prematurity (ROP).***

**Table of Contents**

**I. Periventricular leukomalacia (PVL)**

• Table S1: Valid datasets for PVL

• Table S2a: χ² tests (<750 g) for categorical variables

• Table S2b: χ² tests (≥750 g) for categorical variables

• Table S3a: Mann–Whitney U tests (<750 g) for continuous variables

• Table S3b: Mann–Whitney U tests (≥750 g) for continuous variables

• Table S4: Complete datasets for full logistic regression

• Table S5: Logistic regression (full model, all prespecified covariates)

• Table S6a: Complete datasets for stepwise forward regression

• Table S6b: Stepwise regression results (<750 g / ≥750 g)

• Table S7a: Complete datasets for reduced 5-variable regression

• Table S7b: Reduced model results (<750 g / ≥750 g)

**II. Retinopathy of prematurity (ROP)**

• Table S8: Distribution of ROP stages (0–5)

• Table S9: Distribution of grouped ROP stages (no ROP vs. severe ROP)

• Table S10a: χ² tests (<750 g) for categorical variables

• Table S10b: χ² tests (≥750 g) for categorical variables

• Table S11a: Mann–Whitney U tests (<750 g) for continuous variables

• Table S11b: Mann–Whitney U tests (≥750 g) for continuous variables

• Table S12: Complete datasets for full logistic regression

• Table S13: Logistic regression (full model, all prespecified covariates)

• Table S14a: Complete datasets for stepwise forward regression

• Table S14b: Stepwise regression results (<750 g / ≥750 g)

• Table S15a: Complete datasets for reduced 5-variable regression

• Table S15b: Reduced model results (<750 g / ≥750 g)

**Abbreviations:**

PVL = Periventricular leukomalacia

RBC = Red blood cell

EPO = Erythropoietin

Apgar 10 = Apgar score at 10 minutes

Days on oxygen = Duration of oxygen therapy

End of any respiratory support = Day of life at termination of respiratory support

Sectio = Cesarean section

Emergency sectio = Emergency cesarean section

Max. O₂ = Maximum oxygen requirement in the first 12 hours of life

NIPPV = Non-invasive intermittent positive pressure ventilation (CPAP with intermittent positive pressure breaths)

CPAP = Continuous positive airway pressure

Any CPAP = Any use of CPAP

Twin-to-twin transfusion = Twin-to-twin transfusion syndrome

**I. Periventricular leukomalacia (PVL)**

**A. Univariate analyses**

**Table S1: Valid datasets for PVL:** Number of valid and missing PVL datasets in the GNN cohort.

|  | n | % |
| --- | --- | --- |
| Valid data | 12198 | 99,6 |
| Missing data | 52 | 0.4 |
| Total | 12250 | 100 |
|  |  |  |

*Percentages are calculated per available cases. Deviations between the stated N and the sum of categories reflect missing data. Minor deviations in percentages are due to rounding. Statistical significance was defined as p<0.05 (two-sided). PVL status was documented in 12 198 infants (99.6% of the cohort). Only 52 infants (0.4%) had missing data.*

**Table S2a: Chi² tests (<750 g):** Results of χ² tests for categorical variables in infants <750 g BW.

| **Variables** |  | **Category** |  |  | **PVL** |  |  |  | **Significance** |
| --- | --- | --- | --- | --- | --- | --- | --- | --- | --- |
|  | **N** |  | **n** | **%** | **0** | **%** | **1** | **%** |  |
| Sex | 2072 | Male | 950 | 45.8 | 900 | 94.7 | 50 | 5.3 |  |
|  |  | Female | 1122 | 54.2 | 1076 | 95.9 | 46 | 4.1 | 0.209 |
| Multiple birth | 2067 | Yes | 1554 | 75.2 | 1478 | 95.1 | 76 | 4.9 |  |
|  |  | No | 513 | 24.8 | 493 | 96.1 | 20 | 3.9 | 0.355 |
| Mode of delivery | 1954 | Spontaneous | 176 | 10.6 | 164 | 93.2 | 12 | 6.8 |  |
|  |  | Sectio | 1305 | 78.5 | 1252 | 95.9 | 53 | 4.1 |  |
|  |  | Emergency sectio | 182 | 10.9 | 168 | 92.3 | 14 | 7.7 | 0.039 |
| Twin-to-twin transfusion | 1975 | No | 1896 | 97.0 | 1805 | 95.2 | 91 | 4.8 |  |
|  |  | Yes | 58 | 3.0 | 56 | 96.6 | 2 | 3.4 | 0.634 |
| RBC transfusion | 1665 | No | 214 | 12.8 | 209 | 97.7 | 5 | 2.3 |  |
|  |  | Yes | 1461 | 87.2 | 1387 | 94.9 | 74 | 5.1 | 0.079 |
| Any CPAP | 1675 | No | 3 | 0.2 | 3 | 100 | 0 | 0.0 |  |
|  |  | Yes | 1672 | 99.8 | 1593 | 95.3 | 79 | 4.7 | 0.700 |
| NIPPV | 1268 | No | 570 | 45.0 | 542 | 95.1 | 28 | 4.9 |  |
|  |  | Yes | 698 | 55.0 | 663 | 95.0 | 35 | 5.0 | 0.934 |
| CPAP | 1672 | No | 216 | 12.9 | 204 | 94.4 | 12 | 5.6 |  |
|  |  | Yes | 1453 | 86.9 | 1386 | 95.4 | 67 | 4.6 | 0.77 |
| Sepsis | 1817 | No | 842 | 46.3 | 811 | 96.3 | 31 | 3.7 |  |
|  |  | Yes | 975 | 53.7 | 917 | 94.1 | 58 | 5.9 | 0.026 |
| EPO | 2072 | No | 1761 | 85.0 | 1680 | 95.4 | 81 | 4.6 |  |
|  |  | Yes | 311 | 15.0 | 296 | 95.2 | 15 | 4.8 | 0.863 |

*Percentages are calculated per available cases. Deviations between the stated N and the sum of categories reflect missing data. Minor deviations in percentages are due to rounding. Statistical significance was defined as p<0.05 (two-sided).*

**Table S2 b: Chi² tests (≥750 g):** Results of χ² tests for categorical variables in infants ≥750 g BW.

| **Variables** |  | **Category** |  |  | **PVL** |  |  |  | **Significance** |
| --- | --- | --- | --- | --- | --- | --- | --- | --- | --- |
|  | **N** |  | **n** | **%** | **0** | **%** | **1** | **%** |  |
| Sex | 10123 | Male | 5230 | 51.7 | 5114 | 97.8 | 116 | 2.2 |  |
|  |  | Female | 4893 | 48.3 | 4706 | 98.0 | 97 | 2.0 | 0.409 |
| Multiple birth | 10106 | Yes | 6530 | 64.6 | 6303 | 97.9 | 137 | 2.1 |  |
|  |  | No | 3576 | 35.4 | 3501 | 97.9 | 75 | 2.1 | 0.998 |
| Mode of delivery | 7350 | Spontaneous | 5602 | 29.1 | 583 | 97.7 | 14 | 2.3 |  |
|  |  | Sectio | 5973 | 69.3 | 5852 | 98.0 | 121 | 2.0 |  |
|  |  | Emergency sectio | 654 | 8,96 | 629 | 96.2 | 25 | 3.8 | 0.009 |
| Twin-to-twin transfusion | 9004 | No | 8721 | 96.9 | 8540 | 97.9 | 181 | 2.1 |  |
|  |  | Yes | 283 | 3.1 | 270 | 95.4 | 13 | 4.6 | 0.004 |
| RBC transfusion | 7367 | No | 5137 | 69.7 | 5083 | 99.0 | 54 | 1.0 |  |
|  |  | Yes | 2180 | 30.3.6 | 2074 | 95.1 | 106 | 4.9 | <0.0001 |
| Any CPAP | 7135 | No | 394 | 5.5 | 394 | 100.0 | 0 | 0.0 |  |
|  |  | Yes | 6741 | 94.5 | 6581 | 97.6 | 160 | 2.4 | 0.002 |
| NIPPV | 4966 | No | 3619 | 72.9 | 3547 | 98.0 | 72 | 2.0 |  |
|  |  | Yes | 1347 | 27.1 | 1307 | 97.0 | 40 | 3.0 | 0.039 |
| CPAP | 7358 | No | 1097 | 14.9 | 1084 | 98.8 | 13 | 1.2 |  |
|  |  | Yes | 6261 | 85.1 | 6114 | 97.7 | 147 | 2.3 | 0.045 |
| Sepsis | 8329 | No | 6546 | 78.6 | 6446 | 98.5 | 100 | 1.5 |  |
|  |  | Yes | 1783 | 21.4 | 1710 | 95.9 | 73 | 4.1 | <0.0001 |
| EPO | 10126 | No | 9244 | 91.3 | 9048 | 97.9 | 196 | 2.1 |  |
|  |  | Yes | 882 | 8.7 | 865 | 98.1 | 17 | 1.9 | 0.703 |

*Percentages are calculated per available cases. Deviations between the stated N and the sum of categories reflect missing data. Minor deviations in percentages are due to rounding. Statistical significance was defined as p<0.05 (two-sided).*

**Table S3a: Mann–Whitney U tests (<750 g)**: Results of Mann–Whitney U tests for continuous variables in infants BW <750 g BW.

|  | **PVL** | **n** | **Median** | **min** | **max** | **Percentiles** | | | | | | | **Significance** |
| --- | --- | --- | --- | --- | --- | --- | --- | --- | --- | --- | --- | --- | --- |
|  |  |  |  |  |  | **5** | **10** | **25** | **50** | **75** | **90** | **95** |  |
| **Birthweight** | **0** | **1972** | 630 | 240 | 749 | 425 | 475 | 550 | 630 | 700 | 740 |  |  |
|  | **1** | **96** | 620 | 335 | 745 | 409 | 470 | 530 | 620 | 700 | 732 | 740 | 0.639 |
| **Gestational Age** | **0** |  | 25.29 | 22.00 | 34.43 | 23.3 | 23.7 | 24.3 | 25.3 | 26.7 | 28 | 28.9 |  |
|  | **1** |  | 24.79 | 22.71 | 29.43 | 23.2 | 23.6 | 24.1 | 24.8 | 25.6 | 27.9 | 28.4 | 0.003 |
| **Number of multiples** | **0** | **1950** | 1 | 1 | 4 | 1 | 1 | 1 | 1 | 1 | 2 | 2 |  |
|  | **1** | **95** | 1 | 1 | 3 | 1 | 1 | 1 | 1 | 1 | 2 | 2 | 0.372 |
| **RBC transfusions** | **0** | **1587** | 3 | 0 | 31 | 0 | 0 | 1 | 3 | 5 | 8 | 10 |  |
|  | **1** | **79** | 5 | 0 | 34 | 0 | 1 | 3 | 5 | 8 | 11 | **13** | <0.0001 |
| **Days of oxygen therapy** | **0** | **1968** | 51 | 0 | 360 | 0 | 0 | 1 | 51 | 84 | 122 | 148 |  |
|  | **1** | **96** | 70 | 0 | 296 | 0 | **0** | 25 | 70 | 99.3 | 123 | 171 | 0.009 |
| **Max O2** | **0** | **1552** | 40 | 21 | 100 | 21 | 21 | 28 | 40 | 60 | 100 | 100 |  |
|  | **1** | **73** | 40 | 21 | 100 | 21 | 25 | 28 | 40 | 58.5 | 80 | **93** | 0.566 |
| **End of any respiratory support** | **0** | **1577** | 64 | 2 | 420 | 21 | 32 | 48 | 64 | 84 | 129 |  |  |
|  | **1** | **79** | 73 | 6 | 281 | 29 | 35 | 55 | 73 | 100 | 133 | 154 | 0.011 |
| **APGAR 10** | **0** | **1526** | 8 | 2 | 10 | 6 | 7 | 8 |  | 9 | 9 | 10 |  |
|  | **1** | **77** | 8 | 4 | 10 | 5 | **6** | 7 | 8 | 9 | 9 | 10 | 0.028 |

*Percentages are calculated per available cases. Deviations between the stated N and the sum of categories reflect missing data. Minor deviations in percentages are due to rounding. Statistical significance was defined as p<0.05 (two-sided).*

**Table S3b: Mann–Whitney U tests (≥750 g**): Results of Mann–Whitney U tests for continuous variables in infants **≥**750 g BW.

|  | **PVL** | **n** | **Med** | **min** | **max** | **Percentiles** | | | | | | | **Signific.** |
| --- | --- | --- | --- | --- | --- | --- | --- | --- | --- | --- | --- | --- | --- |
|  |  |  |  |  |  | 5 | 10 | 25 | 50 | 75 | 90 | 95 |  |
| **Birthweight** | 0 | 9913 | 1220 | 750 | 1770 | 820 | 870 | 990 | 1220 | 1400 | 1480 | 1490 | 1490 |
|  | 1 | 213 | 1145 | 750 | 1490 | 789 | 820 | 913 | 1145 | 1328 | 1460 | 1480 | <0.0001 |
| **Gestational Age** | 0 | 9906 | 29.6 | 22.7 | 36.9 | 26 | 26.7 | 28.1 | 29.9 | 31.4 | 32.9 | 33.9 |  |
|  | 1 | 213 | 28.3 | 24.3 | 35.2 | 25 | 25.6 | 26.8 | 28.3 | 29.9 | 31.6 | 32.3 | <0.0001 |
| **Number of multiples** | 0 | 9706 | 1 | 1 | 5 | 1 | 1 | 1 | 1 | 2 | 2 | 3 |  |
|  | 1 | 210 | 1 | 1 | 4 | 1 | 1 | 1 | 1 | 2 | 2 | 2 | 0.958 |
| **RBC transfusions** | 0 | 7196 | 0 | 0 | 22 | 0 | 0 | 0 | 0 | 1 | 2 | 3 |  |
|  | 1 | 159 | 1 | 0 | 33 | 0 | 0 | 0 | 1 | 3 | 6 | 8 | <0.0001 |
| **Days of oxygen therapy** | 0 | 9897 | 1 | 0 | 249 | 0 | 0 | 0 | 1 | 5 | 38 | 56 |  |
|  | 1 | 211 | 3 | 0 | 214 | 0 | 0 | 0 | 3 | 39 | 76 | 98.2 | <0.0001 |
| **Max O2** | 0 | 7036 | 30 | 21 | 100 | 21 | 21 | 21 | 30 | 40 | 60 | 88 |  |
|  | 1 | 157 | 40 | 21 | 100 | 21 | 21 | 27 | 40 | 60 | 99.2 | 100 | <0.0001 |
| **End of any respiratory support** | 0 | 6559 | 15 | 0 | 249 | 2 | 2 | 5 | 15 | 34 | 51 | 63 |  |
|  | **1** | 159 | 31 | 1 | 207 | 3 | 5 | 12 | 31 | 55 |  | 85 | <0.0001 |
| **APGAR 10** | **0** | 806 | 9 | 0 | 10 | **7** | 8 | 8 | 9 | 9 | 10 | 10 |  |
|  | **1** | 151 | 8 | 1 | 10 | 7 | 7 | 8 | 8 | 9 | **9** | 10 | <0.001 |

*Percentages are calculated per available cases. Deviations between the stated N and the sum of categories reflect missing data. Minor deviations in percentages are due to rounding. Statistical significance was defined as p<0.05 (two-sided).*

**B. Multivariate models**

**Table S4: Distribution of variables** Number of complete datasets available for the full logistic regression model.

| **Weight group** | **Observed** |  | **Predicted** | |
| --- | --- | --- | --- | --- |
|  |  |  | **PVL** | |
|  |  |  | **0** | **1** |
| <750g | PVL | 0 | 1412 | 0 |
|  |  | 1 | 69 | 0 |
| ≥750g | PVL | 0 | 5944 | 0 |
|  |  | 1 | 140 | 0 |

**Table S5: Logistic regression (full model):** Results of the full model including all prespecified covariates.

| **Weight group** | **Step** | **Variable** | **Significance** | **OR** |
| --- | --- | --- | --- | --- |
| <750g | 1 | RBC transfusions | <0.0001 | 1.142 |
|  | 2 | Sepsis | 0.018 | 1.983 |
|  |  | RBC transfusions | <0.0001 | 1.123 |
|  | 3 | Spontaneous | 0.045 |  |
|  |  | Sectio | 0.209 | 0.635 |
|  |  | Emergency sectio | 0.426 | 1.411 |
|  |  | Sepsis | 0.015 | 1.938 |
|  |  | RBC transfusions | <0.0001 | 1.117 |
|  | | | | |
| ≥750g | 1 | RBC transfusions | <0.0001 | 1.298 |
|  | 2 | RBC transfusions | <0.0001 | 1.276 |
|  |  | Max. O₂ | <0.0001 | 1.014 |
|  | 3 | RBC transfusions | <0.0001 | 1.237 |
|  |  | Max. O₂ | 0.001 | 1.012 |
|  |  | Gestational age | 0.003 | 0.874 |
|  | 4 | RBC transfusions | <0.0001 | 1.253 |
|  |  | Max. O₂ | 0.002 | 1.011 |
|  |  | Gestational age | <0.0001 | 0.782 |
|  |  | Birth weight | 0.002 | 1.002 |
|  | 5 | RBC transfusions | <0.0001 | 1.262 |
|  |  | Max. O₂ | 0.039 | 1.012 |
|  |  | Gestational age | <0.0001 | 0.764 |
|  |  | Birth weight | 0.003 | 1.002 |
|  |  | Sex | 0.009 | 0.616 |
|  | 6 | RBC transfusions | <0.001 | 1.259 |
|  |  | Max. O₂ | 0.006 | 1.01 |
|  |  | Gestational age | <0.0001 | 0.78 |
|  |  | Birth weight | 0.004 | 1.002 |
|  |  | Sex | 0.008 | 0.622 |
|  |  | APGAR 10 | 0.03 | 0.851 |
|  | 7 | RBC transfusions | <0.0001 | 1.259 |
|  |  | Max. O₂ | 0.006 | 1.01 |
|  |  | Gestational age | <0.0001 | 0.775 |
|  |  | Birth weight | 0.003 | 1.002 |
|  |  | Sex | 0.009 | 0.628 |
|  |  | APGAR 10 | 0.032 | 0.852 |
|  |  | Twin-to-twin transfusion | 0.051 | 1.952 |

**C. Stepwise regression**

**Table S6a: Available datasets Stepwise:** Number of complete datasets available for stepwise forward regression.

| Weight group | Observed |  | Predicted | |
| --- | --- | --- | --- | --- |
|  |  |  | **PVL** |  |
|  |  |  | **0** | **1** |
| **<750g** | **PVL** | **0** | 1507 | 0 |
|  |  | **1** | 77 | 0 |
| **≥750g** | **PVL** | **0** | 6942 | 3 |
|  |  | **1** | 148 | 2 |

**Table S6b: Results Stepwise (<750 g / ≥750 g):** Results of the stepwise forward regression in both weight strata.

| **Weight group** | **Step** | **Variable** | **Significance** | **OR** |
| --- | --- | --- | --- | --- |
| <750g | 1 | Spontaneous | 0.124 |  |
|  |  | Sectio | 0.286 | 0.693 |
|  |  | Emergency sectio | 0.558 | 1.278 |
|  |  | Sepsis | 0.082 | 1.571 |
|  |  | RBC transfusions | <0.0001 | 1.104 |
|  |  | APGAR 10 | 0.148 | 0.884 |
|  | | | | |
| ≥750g | 1 | Spontaneous | 0.077 |  |
|  |  | Sectio | 0.88 | 1.048 |
|  |  | Emergency sectio. | 0.12 | 1.758 |
|  |  | Sepsis | 0.004 | 1.675 |
|  |  | RBC transfusions | <0.0001 | 1.262 |
|  |  | APGAR 10 | <0.0001 | 0.77 |

**Table S7a: Available datasets Reduced Model:** Results of the reduced model including five key predictors (RBC transfusions. gestational age. birth weight. oxygen exposure. sepsis).

| **Weight group** | **Observed** |  | **Predicted** | |
| --- | --- | --- | --- | --- |
|  |  |  | **PVL** |  |
|  |  |  | **0** | **1** |
| <750g | PVL | 0 | 1507 | 0 |
|  |  | 1 | 77 | 0 |
| ≥750g | PVL | 0 | 6942 | 3 |
|  |  | 1 | 148 | 2 |

**Table S7b: Results Reduced Model (<750 g / ≥750 g):** Results of the reduced model including five key predictors (RBC transfusions. gestational age. birth weight. oxygen exposure. sepsis).

| **Weight group** | **Step** | **Variable** | **Significance** | **OR** |
| --- | --- | --- | --- | --- |
| <750g | 1 | RBC transfusions | <0.0001 | 1.142 |
|  | 2 | Sepsis | 0.018 | 1.983 |
|  |  | RBC transfusions | <0.0001 | 1.123 |
|  | 3 | Spontaneous | 0.036 |  |
|  |  | Sectio | 0.209 | 0.635 |
|  |  | Emergency sectio | 0.426 | 1.411 |
|  |  | Sepsis | 0.022 | 1.938 |
|  |  | RBC transfusions | <0.0001 | 1.117 |
|  | | | | |
| ≥750g | 1 | RBC transfusions | <0.0001 | 1.298 |
|  | 2 | RBC transfusions | <0.0001 | 1.276 |
|  |  | Max. O₂ | <0.0001 | 1.014 |
|  | 3 | RBC transfusions | <0.0001 | 1.237 |
|  |  | Max. O₂ | 0.001 | 1.012 |
|  |  | Gestational age | 0.003 | 0.874 |
|  | 4 | RBC transfusions | <0.0001 | 1.253 |
|  |  | Max. O₂ | 0.002 | 1.011 |
|  |  | Gestational age | <0.0001 | 0.782 |
|  |  | Birth weight | 0.007 | 1.002 |
|  | 5 | RBC transfusions | <0.0001 | 1.262 |
|  |  | Max. O₂ | 0.001 | 1.012 |
|  |  | Gestational age | <0.0001 | 0.764 |
|  |  | Birth weight | 0.003 | 1.002 |
|  |  | Sex | 0.007 | 0.616 |
|  | 6 | RBC transfusions | <0.001 | 1.259 |
|  |  | Max. O₂ | 0.006 | 1.01 |
|  |  | Gestational age | <0.0001 | 0.78 |
|  |  | Birth weight | 0.004 | 1.002 |
|  |  | Sex | 0.008 | 0.622 |
|  |  | APGAR 10 | 0.03 | 0.851 |
|  | 7 | RBC transfusions | <0.0001 | 1.259 |
|  |  | Max. O₂ | 0.006 | 1.01 |
|  |  | Gestational age | <0.0001 | 0.775 |
|  |  | Birth weight | 0.003 | 1.002 |
|  |  | Sex | 0.009 | 0.628 |
|  |  | APGAR 10 | <0.0001 | 0.852 |
|  |  | Twin-to-twin transfusion | 0.051 | 1.952 |

* Max. oxygen requirement in the first 12 hours of life

**II Retinopathy of prematurity (ROP).**

**A. Univariate analyses**

**S8: Distribution of ROP stages (0–5**) Number of infants by ROP stage (0–5).

|  | **ROP stages** | **n** | **%** |
| --- | --- | --- | --- |
| Valid data | 0 | 5375 | 43.9 |
|  | 1 | 1315 | 10.7 |
|  | 2 | 849 | 6.9 |
|  | 3 | 423 | 3.5 |
|  | 4 | 10 | 0.1 |
|  | 5 | 3 | 0 |
|  | Total | 7975 | 65.1 |
| Missing data | Total | 4275 | 34.9 |
| Total |  | 12250 | 100 |

*Percentages are calculated per available cases. Deviations between the stated N and the sum of categories reflect missing data. Minor deviations in percentages are due to rounding. Statistical significance was defined as p<0.05 (two-sided).*

**S9: Distribution of grouped ROP stages (no ROP vs sROP = stages 3–5)** Number of infants with no ROP vs severe ROP (sROP).

|  | **sROP** | **n** | **%** |
| --- | --- | --- | --- |
| Valid data | 0 | 7539 | 61.5 |
|  | 1 | 436 | 3.6 |
|  | Total | 7975 | 65.1 |
| Missing data | Total | 4275 | 34.9 |
| Total |  | 12250 | 100 |

*Percentages are calculated per available cases. Deviations between the stated N and the sum of categories reflect missing data. Minor deviations in percentages are due to rounding. Statistical significance was defined as p<0.05 (two-sided).* *Valid ROP staging was available in 7 975 infants (65.1% of the cohort). Missing data (n=4 275; 34.9%) were mainly due to incomplete ophthalmologic documentation.*

**S10a: χ² tests (<750 g)** Results of χ² tests for categorical variables b

| **Variables** |  | **Category** |  |  | **sROP** |  |  |  | **Significance** |
| --- | --- | --- | --- | --- | --- | --- | --- | --- | --- |
|  | **N** |  | **n** | **%** | **0** | **%** | **1** | **%** |  |
| Sex | 1630 | Male | 749 | 46.0 | 585 | 78.1 | 164 | 21.9 |  |
|  |  | Female | 881 | 54.0 | 730 | 82.9 | 151 | 17.1 | 0.015 |
| Multiple birth | 1629 | Yes | 1217 | 74.7 | 990 | 81.3 | 227 | 18.7 |  |
|  |  | No | 412 | 25.3 | 325 | 78.9 | 87 | 21.1 | 0.273 |
| Mode of delivery | 1618 | Spontaneous | 175 | 10.8 | 123 | 70.3 | 52 | 29.7 |  |
|  |  | Sectio | 1264 | 78.1 | 1049 | 83.0 | 215 | 17.0 |  |
|  |  | Emergency sectio | 179 | 11.1 | 136 | 76.0 | 43 | 24.0 | <0.0001 |
| Twin-to-twin transfusion | 1603 | No | 1547 | 96.5 | 1249 | 80.7 | 298 | 19.3 |  |
|  |  | Yes | 56 | 3.5 | 44 | 78.6 | 12 | 21.4 | 0.687 |
| RBC transfusion | 1630 | No | 206 | 12.6 | 193 | 93.7 | 13 | 6.3 |  |
|  |  | Yes | 1424 | 87.4 | 1127 | 78.8 | 302 | 21.2 | <0.0001 |
| Any CPAP | 1630 | No | 2 | 0.1 | 2 | 100.0 | 0 | 0.0 |  |
|  |  | Yes | 1628 | 99.9 | 1313 | 80.7 | 315 | 19.3 | 0.483 |
| NIPPV | 1235 | No | 549 | 44.5 | 433 | 79.8 | 111 | 20.2 |  |
|  |  | Yes | 653 | 55.5 | 537 | 78.3 | 149 | 21.7 | 0.52 |
| CPAP | 1627 | No | 207 | 12.7 | 164 | 79.2 | 43 | 20.8 |  |
|  |  | Yes | 1417 |  | 1147 | 80.9 | 270 | 19.1 | 0.098 |
| Sepsis | 1629 | No | 729 |  | 625 | 85.7 | 104 | 14.3 |  |
|  |  | Yes | 900 | 55.2 | 689 | 76.6 | 211 | 23.4 | <0.0001 |
| EPO | 1630 | No | 1327 | 81.4 | 1053 | 79.4 | 274 | 20.6 |  |
|  |  | Yes | 303 | 18.6 | 262 | 86.5 | 41 | 13.5 | 0.005 |

*Percentages are calculated per available cases. Deviations between the stated N and the sum of categories reflect missing data. Minor deviations in percentages are due to rounding. Statistical significance was defined as p<0.05 (two-sided).*

**S10b: χ² tests (≥750 g)** Results of χ² tests for categorical variables in infants ≥750 g BW

| **Variables** |  | **Category** |  |  | **sROP** |  |  |  | **Significance** |
| --- | --- | --- | --- | --- | --- | --- | --- | --- | --- |
|  | **N** |  | **n** | **%** | **0** | **%** | **1** | **%** |  |
| Sex | 6345 | Male | 3318 | 52.3 | 3250 | 98.0 | 63 | 2.0 |  |
|  |  | Female | 3027 | 47.7 | 2974 | 98.2 | 53 | 1.8 | 0.335 |
| Multiple birth | 6344 | Yes | 4017 | 63.3 | 3936 | 98.0 | 8 | 2.0 |  |
|  |  | No | 2327 | 36.7 | 2287 | 93.3 | 40 | 1.7 | 0.404 |
| Mode of delivery | 6317 | Spontaneous | 530 | 8.4 | 514 | 97.0 | 16 | 3.0 |  |
|  |  | Sectio | 5204 | 82.4 | 5116 | 98.3 | 88 | 1.7 |  |
|  |  | Emergency sectio | 583 | 9.2 | 566 | 97.1 | 17 | 2.9 | 0.019 |
| Twin-to-twin transfusion | 6147 | No | 5903 | 96.0 | 5787 | 98.0 | 116 | 2.0 |  |
|  |  | Yes | 244 | 4.0 | 240 | 98.4 | 4 | 1.6 | 0.719 |
| RBC transfusion | 6333 | No | 4276 | 67.5 | 4252 | 99.4 | 24 | 0.6 |  |
|  |  | Yes | 2057 | 32.5 | 1960 | 95.3 | 97 | 4.7 | <0.0001 |
| Any CPAP | 6155 | No | 204 | 3.3 | 204 | 100.0 | 0 | 0.0 |  |
|  |  | Yes | 5951 | 96.7 | 5330 | 98.0 | 021 | 2.0 | 0.04 |
| NIPPV | 4204 | No | 2955 | 70.3 | 2913 | 93.6 | 42 | 1.4 |  |
|  |  | Yes | 1249 | 29.7 | 1215 | 97.3 | 34 | 2.7 | 0.004 |
| CPAP | 6330 | No | 828 | 13.1 | 821 | 99.2 | 7 | 0.8 |  |
|  |  | Yes | 5498 | 86.9 | 5384 | 97.9 | 114 | 2.1 | 0.053 |
| Sepsis | 6340 | No | 4842 | 76.4 | 4778 | 98.7 | 64 | 1.3 |  |
|  |  | Yes | 1498 | 23.6 | 1441 | 96.2 | 57 | 3.8 | <0.0001 |
| EPO | 6345 | No | 5559 | 87.6 | 5448 | 98.0 | 111 | 7.0 |  |
|  |  | Yes | 786 | 12.4 | 776 | 98.7 | 10 | 1.3 | 0.165 |

*Percentages are calculated per available cases. Deviations between the stated N and the sum of categories reflect missing data. Minor deviations in percentages are due to rounding. Statistical significance was defined as p<0.05 (two-sided).*

**S11a: Mann–Whitney U tests (<750 g)** Results of Mann–Whitney U tests for continuous variables in infant < 750 g BW

| Variable | sROP | n | Med. | min | max | Percentiles | | | | | | | Signifig. |
| --- | --- | --- | --- | --- | --- | --- | --- | --- | --- | --- | --- | --- | --- |
|  |  |  |  |  |  | 5 | 10 | 25 | 50 | 75 | 90 | 95 |  |
| Birthweight | 0 | 1315 | 640 | 280 | 748 | 430 | 475 | 550 | 640 | 700 | 740 | 740 |  |
|  | 1 | 315 | 600 | 240 | 748 | 380 | 430 | 516 | 600 | 680 | 730 | 740 | < 0.0001 |
| Gestational Age | 0 | 1314 | 25.3 | 22 | 32.4 | 23.4 | 23. | 24.3 | 25.4 | 26.9 | 28.1 | 28.7 |  |
|  | 1 | 315 | 24.6 | 22 | 34.4 | 23 | 23.3 | 23.9 | 24.6 | 25.6 | 26.7 | 27.4 | < 0.0001 |
| Number of multiples | 0 | 1312 | 1 | 1 | 4 | 1 | 1 | 1 | 1 | 1 | 2 | 2 |  |
|  | 1 | 313 | 1 | 1 | 4 | 1 | 1 | 1 | 1 | 2 | 2 | 2 | 0293 |
| RBC transfusions | 0 | 1307 | 3 | 0 | 34ı | 0 | 0 | 1l | 3 | 5 | 7 | 9 |  |
|  | 1 | 314 | 5 | 0 | 31 | 1 | 1 | 3 | 5 | 6 | 11.5 | 14.3 | < 0.0001 |
| Days of oxygen therapy | 0 | 1309 | 58 | 0 | 360 | 1 | 2 | 32 | 56 | 86 | 122 | 148 |  |
|  | 1 | 313 | 93 | 0 | 339 | 21.1 | 40.2 | 63 | 93 | 120 | 160 | 178 | < 0.0001 |
| Max O2 | 0 | 1281 | 39 | 21 | 100 | 21 | 21 | 28 | 39 | 56 | 95.8 | 100 |  |
|  | 1 | 303 | 43 | 21 | 100 | 21 | 25 | 30 | 43 | 70 | 100 | 100 | < 0.0001 |
| End of any respiratory support | 0 | 1301 | 62 | 2 | 420 | 20 | 31 | 46 | 62 | 80 | 101 | 123 |  |
|  | 1 | 312 | 79 | 5 | 281 | 40 | 483 | 63 | 79 | 101 | 132 | 161 | < 0.0001 |
| APGAR 10 | 0 | 1257 | 8 | 2 | 10 | 6 | 7 | 8 | 8 | 9 | 9 | 10 |  |
|  | 1 | 303 | 8 | 2 | 10 | 5 | 7 | 7 | 8 | 9 | 9 | 9 | 0.011 |

*Percentages are calculated per available cases. Deviations between the stated N and the sum of categories reflect missing data. Minor deviations in percentages are due to rounding. Statistical significance was defined as p<0.05 (two-sided).*

**S11b: Mann–Whitney U tests (≥750 g)** Results of Mann–Whitney U tests for continuous variables in infant ≥750 g BW

| **Variable** | **sROP** | **N** | **Med** | **min** | **max** | **Percentiles** | | | | | | | **Signifig.** |
| --- | --- | --- | --- | --- | --- | --- | --- | --- | --- | --- | --- | --- | --- |
|  |  |  |  |  |  | 5 | 10 | 25 | 50 | 75 | 90 | 95 |  |
| Birthweight | 0 | 6224 | 1180 | 750 | 1470 | 810 | 855 | 980 | 1180 | 1360 | 1460 | 1483 |  |
|  | 1 | 121 | 900 | 750 | 1436 | 770 | 781 | 833 | 900 | 990 | 1128 | 1317 | < 0.0001 |
| Gestational Age | 0 | 6223 | 29.43 | 23.5 | 36.9 | 25.9 | 26.6 | 27.9 | 29.4 | 30.4 | 32 | 32.9 |  |
|  | 1 | 120 | 26.36 | 23.5 | 33.4 | 24 | 24.6 | 25.6 | 26.4 | 27.7 | 29.3 | 30.1 | < 0.0001 |
| Number of multiples | 0 | 6213 | 1 | 1 | 5 | 1 | 1 | 1 | 1 | 2 | 2 | 3 |  |
|  | 1 | 121 | 1 | 1 | 3 | 1 | 1 | 1 | 1 | 2 | 2 | 2 | 0.312 |
| RBC transfusions | 0 | 6200 | 0 | 0 | 33 | 0 | 0. | 0l | 0 | 1 | 2 | 3 |  |
|  | 1 | 120 | 3 | 0 | 14 | 0 | 0 | 1 | 3 | 4.75 | 7.9 | 11 | < 0.0001 |
| Days of oxygen therapy | 0 | 6209 | 3 | 0 | 218 | 0 | 0 | 1 | 3 | 21 | 48 | 65 |  |
|  | 1 | 121 | 54 | 0 | 214 | 1 | 1 | 17.5 | 54 | 75 | 104 | 142 | < 0.0001 |
| Max O2 | 0 | 6082 | 30 | 21 | 100 | 21 | 21 | 21 | 30 | 40 | 63 | 90 |  |
|  | 1 | 116 | 40 | 21 | 100 | 21 | 21 | 29.3 | 40 | 60 | 100 | 100 | < 0.0001 |
| End of any respiratory support | 0 | 5808 | 17 | 0\| | 186 | 2 | 3 | 6 | 17 | 36 | 52 | 6 |  |
|  | 1 | 120 | 52.5 | 1 | 207 | 7.05 | 14.1 | 33\| | 52.5 | 72.7 | 93.7 | 120 | <0. 0001 |
| APGAR 10 | 0 | 6013 | 9 | 0 | 10 | F | 8 | 8 | 9 | 9 | 10 | 10 |  |
|  | 1 | 120 | 8 | 1 | 107 | 6.05 | 7 | 8 | 8 | 9 | 9 | 10 | <0. 0001 |

*Percentages are calculated per available cases. Deviations between the stated N and the sum of categories reflect missing data. Minor deviations in percentages are due to rounding. Statistical significance was defined as p<0.05 (two-sided).*

**B. Multivariate models**

**S12: Distribution of variables for logistic regression** Number of complete datasets available for the full logistic regression model.

| Weight group | Observed | | Predicted | |
| --- | --- | --- | --- | --- |
|  |  | | sROP | |
|  |  |  | 0 | 1 |
| <750g | sROP | 0 | 1169 | 0 |
|  |  | 1 | 275 | 0 |
| ≥750g | sROP | 0 | 5249 | 0 |
|  |  | 1 | 111 | 0 |

**S12: Logistic regression (full model)** Results of the full model including all prespecified covariates.

| **Weight group** | **Step** | **Variable** | **P value** | **OR** |
| --- | --- | --- | --- | --- |
| <750g | 1 | RBC transfusions | <0.0001 | 1.188 |
|  | 2 | RBC transfusions | <0.0001 | 1.126 |
|  |  | Days of oxygen therapy | <0.0001 | 1.009 |
|  | 3 | Gestational age | <0.0001 | 0.797 |
|  |  | RBC transfusions | <0.0001 | 1.099 |
|  |  | Days of oxygen therapy | <0.0001 | 1.008 |
|  | 4 | Sectio | 0.033 | 0.636 |
|  |  | Emergency sektio | 0.744 | 0.914 |
|  |  | Gestational age | <0.0001 | 0.821 |
|  |  | RBC transfusions | <0.0001 | 1.098 |
|  |  | Days of oxygen therapy | <0.0001 | 1.008 |
| ≥750g | 1 | RBC transfusions | <0.0001 | 1.358 |
|  | 2 | RBC transfusions | <0.0001 | 1.208 |
|  |  | Gestational age | <0.0001 | 0.549 |
|  | 3 | Gestational age | <0.0001 | 0.624 |
|  |  | RBC transfusions | <0.0001 | 1.149 |
|  |  | End of respiratory support (days of life) | <0.0001 | 1.018 |

**C. Stepwise regression**

**S13a: Available datasets for stepwise regression** Number of complete datasets available for stepwise forward regression.

| **Weight group** | **Observed** | | **Predicted** | |
| --- | --- | --- | --- | --- |
|  |  |  | sROP |  |
|  |  |  | 0 | 1 |
| <750g | sROP | 0 | 1279 | 20 |
|  |  | 1 | 286 | 26 |
| ≥750g | sROP | 0 | 6177 | 2 |
|  |  | 1 | 117 | 2 |

**S13b: Results stepwise regression (<750 g / ≥750 g)** Results of the stepwise forward regression in both weight strata.

| **Weight group** | **Step** | **Variable** | **P value** | **OR** |
| --- | --- | --- | --- | --- |
| <750g | 1 | Sepsis | 0.107 | 1.258 |
|  |  | Birth weight | 0.62 | 1.000 |
|  |  | Gestational age | <0.0001 | 0.819 |
|  |  | RBC transfusions | <0.0001 | 1.092 |
|  |  | Days of oxygen therapy | <0.0001 | 1.008 |
| ≥750g | 1 | Sepsis | 0.632 | 1.103 |
|  |  | Birth weight | 0.26 | 0.999 |
|  |  | Gestational age | <0.0001 | 0.634 |
|  |  | RBC transfusions | <0.0001 | 1.148 |
|  |  | Days of oxygen therapy | <0.0001 | 1.012 |

**S14a: Available datasets for reduced model** Number of complete datasets available for the reduced logistic regression model.

| Weight group | Observed | | Predicted | |
| --- | --- | --- | --- | --- |
|  |  |  | sROP |  |
|  |  |  | 0 | 1 |
| <750 g | sROP | 0 | 1279 | 20 |
|  |  | 1 | 286 | 26 |
| ≥750 g | sROP | 0 | 6177 | 2 |
|  |  | 1 | 117 | 2 |

**S14b: Results reduced model (<750 g / ≥750 g**) Results of the reduced model including five key predictors (RBC transfusions, gestational age, birth weight, oxygen exposure, sepsis).

| Weight group | Step | Variable | P value | OR |
| --- | --- | --- | --- | --- |
| <750 g | 1 | Sepsis | 0.107 | 1.258 |
|  |  | Birth weight | 0.62 | 1.000 |
|  |  | Gestational age | <0.0001 | 0.819 |
|  |  | RBC transfusions | <0.0001 | 1.092 |
|  |  | Days of oxygen therapy | <0.0001 | 1.008 |
| ≥750 g | 1 | Sepsis | 0.632 | 1.103 |
|  |  | Birth weight | 0.26 | 0.999 |
|  |  | Gestational age | <0.0001 | 0.634 |
|  |  | RBC transfusions | <0.0001 | 1.148 |
|  |  | Days of oxygen therapy | <0.0001 | 1.012 |
