# Supplementary material for: Association of red blood cell transfusions with periventricular leukomalacia in very preterm infants
Source: Vox Sang. 2026 Mar 12;121(6):842–7. doi: 10.1111/vox.70239 (PMC13253060; doi:10.1111/vox.70239)
Supplement: Supplementary file 2 — Supplementary 2 German Neonatal Network (GNN) Collaborators (2009–2015). [file VOX-121-842-s002.docx]

# Supplement 2 – German Neonatal Network (GNN) Collaborators (2009–2015)

The following investigators and centers of the German Neonatal Network (GNN) contributed to data collection and clinical care for very preterm infants during the study period 2009–2015.

| Name | Institution | City | Country |
| --- | --- | --- | --- |
| Egbert Herting | Paediatrics, University of Lübeck | Lübeck | Germany |
| Wolfgang Göpel | Paediatrics, University of Lübeck | Lübeck | Germany |
| Alexander Humberg | Paediatrics, University of Lübeck | Lübeck | Germany |
| Christoph Härtel | Paediatrics, University of Lübeck | Lübeck | Germany |
| Guido Stichtenoth | Paediatrics, University of Lübeck | Lübeck | Germany |
| Philipp Jung | Paediatrics, University of Lübeck | Lübeck | Germany |
| Tanja K Rausch | Institute for Medical Biometry and Statistics Lübeck | Lübeck | Germany |
| Thorsten Orlikowsky | Neonatology, University of Aachen | Aachen | Germany |
| Christian Wieg | Children's Hospital Aschaffenburg-Alzenau | Aschaffenburg | Germany |
| Rainer Rossi | Vivantes Klinikum Neukölln | Berlin | Germany |
| Ursula Weller | Department of Paediatrics, Evangelical Klinikum Bethel | Bielefeld | Germany |
| Norbert Teig | Paediatrics, University Hospital | Bochum | Germany |
| Katja Schneider | Department of Paediatrics, GFO Hospitals Bonn | Bonn | Germany |
| Hubert Gerleve | Department of Paediatrics, Christophorus Kliniken Coesfeld | Coesfeld | Germany |
| Angela Kribs | Neonatology and Pediatric Intensive Care, University Hospital of Cologne | Cologne | Germany |
| Claudia Roll | Neonatology and Paediatric Intensive Care, Vest Children's Hospital Datteln, University Witten-Herdecke | Datteln | Germany |
| Thomas Brune | Department of Pediatrics, Klinikum Lippe | Detmold | Germany |
| Friedhelm Heitmann | Department of Paediatrics, Klinikum Dortmund | Dortmund | Germany |
| Michael Mögel | Department of Neonatology and Pediatric Intensive Care, University Hospital Carl Gustav Carus | Dresden | Germany |
| Thomas Höhn | Department of Paediatrics, University of Düsseldorf | Düsseldorf | Germany |
| Ursula Felderhoff-Müser | Department of Neonatology, University Hospital of Essen | Essen | Germany |
| Michael Dördelmann | Department of Paediatrics, Diakonissen Hospital Flensburg | Flensburg | Germany |
| Silke Ehlers | Department of Neonatology, Bürgerhospital Frankfurt | Frankfurt | Germany |
| Knud Linnemann | Department of Paediatrics, University of Greifswald | Greifswald | Germany |
| Roland Haase | Children's Hospital, University of Halle | Halle/Saale | Germany |
| Axel von der Wense | Department of Neonatology, Children's Hospital Hamburg-Altona | Hamburg | Germany |
| Susanne Schmidtke | Department of Neonatology, Asklepios Hospital Hamburg-Barmbek | Hamburg-Barmbek | Germany |
| Bettina Bohnhorst | Pediatric Pulmonology and Neonatology, Hannover Medical School | Hannover | Germany |
| Reinhard Jensen | Department of Paediatrics, Westküstenklinikum Heide | Heide | Germany |
| Michael Zemlin | General Pediatrics and Neonatology, Saarland University | Homburg/Saar | Germany |
| Georg Hillebrand | Department of Paediatrics, Hospital Itzehoe | Itzehoe | Germany |
| Kai Böckenholt | Children's Hospital of the City of Cologne | Köln | Germany |
| Joachim G Eichhorn | Department of Paediatrics, Klinikum Leverkusen gGmbH | Leverkusen | Germany |
| Ralf Böttger | Department of Neonatology, Universitatsklinikum Magdeburg | Magdeburg | Germany |
| Thomas Schaible | Department of Paediatrics, University Medical Center Mannheim | Mannheim | Germany |
| Jürgen Wintgens | Department of Paediatrics, Hospital Mönchengladbach | Mönchengladbach | Germany |
| Claudius Werner | Department of Paediatrics, University of Münster | Münster | Germany |
| Stefan Schäfer | Children's Hospital (Städtisches Klinikum) Nürnberg | Nürnberg | Germany |
| Hugo Segerer | Neonatology, Krankenhaus Barmherzige Brüder | Regensburg | Germany |
| Dirk M Olbertz | Department of Neonatology, Klinikum Südstadt Rostock | Rostock | Germany |
| Jens Möller | Department of Paediatrics, Saarbrücken General Hospital | Saarbrücken | Germany |
| Olaf Kannt | Helios Klinik Schwerin | Schwerin | Germany |
| Mechthild Hubert | Department of Neonatology and Pediatric Intensive Care, DRK Children's Hospital | Siegen | Germany |
| Matthias Vochem | Department of Neonatology, Olgahospital Stuttgart | Stuttgart | Germany |
| Axel Franz | Neonatology, University of Tübingen | Tübingen | Germany |
| Michael Heldmann | HELIOS Children's Hospital Wuppertal, Witten/Herdecke University | Wuppertal | Germany |
